# Complete genome sequence of the thermophilic sulfur-reducer *Desulfurobacterium thermolithotrophum* type strain (BSA^T^) from a deep-sea hydrothermal vent

**DOI:** 10.4056/sigs.2465574

**Published:** 2011-12-30

**Authors:** Markus Göker, Hajnalka Daligault, Romano Mwirichia, Alla Lapidus, Susan Lucas, Shweta Deshpande, Ioanna Pagani, Roxanne Tapia, Jan-Fang Cheng, Lynne Goodwin, Sam Pitluck, Konstantinos Liolios, Natalia Ivanova, Konstantinos Mavromatis, Natalia Mikhailova, Amrita Pati, Amy Chen, Krishna Palaniappan, Cliff Han, Miriam Land, Loren Hauser, Chongle Pan, Evelyne-Marie Brambilla, Manfred Rohde, Stefan Spring, Johannes Sikorski, Reinhard Wirth, John C. Detter, Tanja Woyke, James Bristow, Jonathan A. Eisen, Victor Markowitz, Philip Hugenholtz, Nikos C. Kyrpides, Hans-Peter Klenk

**Affiliations:** 1 Leibniz Institute DSMZ - German Collection of Microorganisms and Cell Cultures, Braunschweig, Germany; 2 Los Alamos National Laboratory, Bioscience Division, Los Alamos, New Mexico, USA; 3 Jomo Kenyatta University of Agriculture and Technology, Nairobi, Kenya; 4 DOE Joint Genome Institute, Walnut Creek, California, USA; 5 Biological Data Management and Technology Center, Lawrence Berkeley National Laboratory, Berkeley, California, USA; 6 Oak Ridge National Laboratory, Oak Ridge, Tennessee, USA; 7 HZI – Helmholtz Centre for Infection Research, Braunschweig, Germany; 8 University of Regensburg, Microbiology – Archaeenzentrum, Regensburg, Germany; 9 University of California Davis Genome Center, Davis, California, USA; 10 Australian Centre for Ecogenomics, School of Chemistry and Molecular Biosciences, The University of Queensland, Brisbane, Australia

**Keywords:** anaerobic, thermophilic, neutrophilic, obligately chemolithoautotrophic, Gram-negative, marine, sulfur-reducing, *Desulfurobacteriaceae*, GEBA

## Abstract

*Desulfurobacterium thermolithotrophum* L'Haridon *et al*. 1998 is the type species of the genus *Desulfurobacterium* which belongs to the family *Desulfurobacteriaceae*. The species is of interest because it represents the first thermophilic bacterium that can act as a primary producer in the temperature range of 45-75 °C (optimum 70°C) and is incapable of growing under microaerophilic conditions. Strain BSA^T^ preferentially synthesizes high-melting-point fatty acids (C_18_ and C_20_) which is hypothesized to be a strategy to ensure the functionality of the membrane at high growth temperatures. This is the second completed genome sequence of a member of the family *Desulfurobacteriaceae* and the first sequence from the genus *Desulfurobacterium*. The 1,541,968 bp long genome harbors 1,543 protein-coding and 51 RNA genes and is a part of the *** G****enomic* ***E****ncyclopedia of* ***B****acteria and* ***A****rchaea * project.

## Introduction

Strain BSA^T^ (= DSM 11699) is the type strain of the species *Desulfurobacterium thermolithotrophum*, which is the type species of its genus *Desulfurobacterium* [1], that currently consists of three validly named species [19]. The genus name is derived from the Latin words '*de*' meaning 'from', 'sulfur', and 'bacterium' meaning 'a stick, staff', yielding the Neo-Latin word 'Desulfurobacterium' meaning 'sulfur-reducing rod-shaped bacterium' [1]. The species epithet is derived from the latinized Greek word 'thermê' meaning 'heat', the latinized Greek word 'lithos' meaning 'stone' and the latinized Greek word 'trophos' meaning 'feeder, rearer, one who feeds', yielding the Neo-Latin word 'thermolithotrophum' meaning 'referring to its thermophilic way of life and lithotrophic metabolism' [1,2]. Strain BSA^T^ was collected from the Snake Pit vent field on the mid Atlantic Ridge with the help of the submersible *Nautile* at a depth of 3,500 m [1]. Although it shares most features with other members of the *Aquificales*, it is distinct in its inability to grow under microaerophilic conditions [1]. Strain BSA^T^ was the first non-hyperthermophilic primary producer isolated from deep-sea vents [1]. Here we present a summary classification and a set of features for *D. thermolithotrophum* strain BSA^T^, together with the description of the complete genomic sequencing and annotation.

## Classification and features

A representative genomic 16S rRNA sequence of *D. thermolithotrophum* BSA^T^ was compared using NCBI BLAST [3,4] under default settings (e.g. considering only the high-scoring segment pairs (HSPs) from the best 250 hits) with the most recent release of the Greengenes database [5] and the relative frequencies of taxa and keywords (reduced to their stem [6] were determined, weighted by BLAST scores. The most frequently occurring genera were *Desulfurobacterium* (30.3%), *Thermoanaerobacter* (18.8%), *Thermovibrio* (14.2%), *Balnearium* (11.0%) and *Persephonella* (4.1%) (80 hits in total). Regarding the two hits to sequences from members of the species, the average identity within HSPs was 98.9%, whereas the average coverage by HSPs was 92.8%. Regarding the single hit to sequences from other members of the genus, the average identity within HSPs was 98.6%, whereas the average coverage by HSPs was 64.4%. Among all other species, the one yielding the highest score was *“Desulfurobacterium crinifex”* (AJ507320), which corresponded to an identity of 98.6% and HSP coverage of 64.4%. (Note that the Greengenes database uses the INSDC (= EMBL/NCBI/DDBJ) annotation, which is not an authoritative source for nomenclature or classification.) The highest-scoring environmental sequence was AF068800 ('hydrothermal vent clone VC2.1Bac24'), which showed an identity of 99.7% and an HSP coverage of 92.7%. The most frequently occurring keywords within the labels of all environmental samples which yielded hits were 'hydrotherm' (5.4%), 'vent' (4.9%), 'microbi' (3.6%), 'water' (2.9%) and 'deep' (2.0%) (167 hits in total). The most frequently occurring keyword within the labels of those environmental samples which yielded hits of a higher score than the highest scoring species was 'hydrotherm, vent' (50.0%) (1 hit in total).

**Figure 1:**
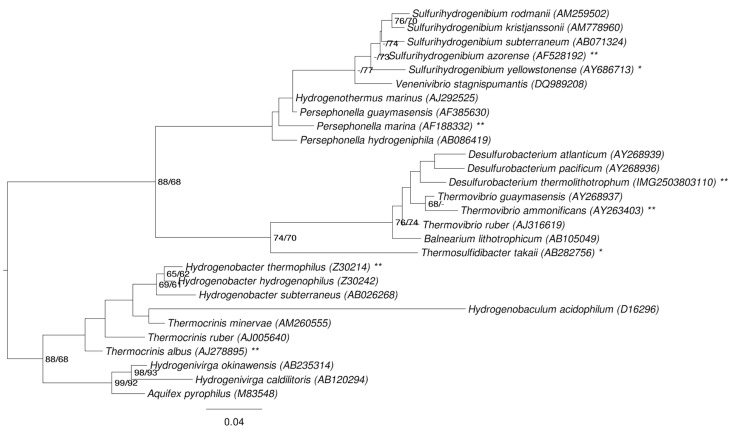
Phylogenetic tree highlighting the position of *D. thermolithotrophum* relative to the type strains of the other species within the order *Aquificales*. The tree was inferred from 1,422 aligned characters [7,8] of the 16S rRNA gene sequence under the maximum likelihood (ML) criterion [9]. Rooting was done initially using the midpoint method [10] and then checked for its agreement with the current classification (Table 1). The branches are scaled in terms of the expected number of substitutions per site. Numbers adjacent to the branches are support values from 1,000 ML bootstrap replicates [11] (left) and from 1,000 maximum parsimony bootstrap replicates [12] (right) if larger than 60%. Lineages with type strain genome sequencing projects registered in GOLD [13] are labeled with one asterisk, those also listed as 'Complete and Published' with two asterisks (referenced in [14-17] and CP002444).

**Figure 1** shows the phylogenetic neighborhood of *D. thermolithotrophum* BSA^T^ in a 16S rRNA based tree. The sequences of the two identical 16S rRNA gene copies in the genome differ by two nucleotides from the previously published 16S rRNA sequence (AJ001049).

**Table 1:** Classification and general *D. thermolithotrophum* BSA^T^ in accordance with the MIGS recommendations [18] and the NamesforLife database [19].

**MIGS ID**	**Property**	**Term**	**Evidence code**
	Current classification	Domain *Bacteria*	TAS [20]
		Phylum ‘*Aquificae*’	TAS [22]
		Class *Aquificae*	TAS [23,24]
		Order *Aquificales*	TAS [23,25,26]
		Family *Desulfurobacteriaceae*	TAS [25]
		Genus *Desulfurobacterium*	TAS [1,25,27]
	Species	*Desulfurobacterium thermolithotrophum*	TAS [1]
MIGS-7	Strain	BSA^T^	TAS [1]
MIGS-12	Reference for biomaterial	DSM 11699	TAS [1]
	Gram stain	negative	TAS [1]
	Cell shape	rod-shaped	TAS [1]
	Motility	motile	TAS [1]
	Sporulation	non-sporulating	TAS [1]
	Temperature range	40-75°C	TAS [1]
	Optimum temperature	70°C	TAS [1]
	Salinity	15 to 70 g per l, optimum at 35 g	TAS [1]
MIGS-22	Oxygen requirement	strictly anaerobic	TAS [1]
	Carbon source	CO_2_	NAS
	Energy metabolism	chemolitoautotrophic, sulfur reduction	TAS [1]
MIGS-6	Habitat	marine	TAS [1]
MIGS-15	Biotic relationship	free-living	TAS [1]
	Biosafety level	1	TAS [28]
MIGS-19	Trophic level	level 1 primary producer	TAS [1]
MIGS-23.1	Isolation	deep-sea hydrothermal vent chimney	TAS [1]
MIGS-4	Geographic location	Snake Pit vent field, Mid-Atlantic Ridge	TAS [1]
MIGS-5	Sample collection time	November/December 1995	TAS [1,29]
MIGS-4.1	Latitude	23.36	TAS [1,29]
MIGS-4.2	Longitude	-44.93	TAS [1,29]
MIGS-4.3	Depth	3,500 m	TAS [1,29]
MIGS-4.4	Altitude	-3,500 m	TAS [1]

Evidence codes - TAS: Traceable Author Statement (i.e., a direct report exists in the literature); NAS: Non-traceable Author Statement (i.e., not directly observed for the living, isolated sample, but based on a generally accepted property for the species, or anecdotal evidence). These evidence codes are from of the Gene Ontology project [30].

**Figure 2:**
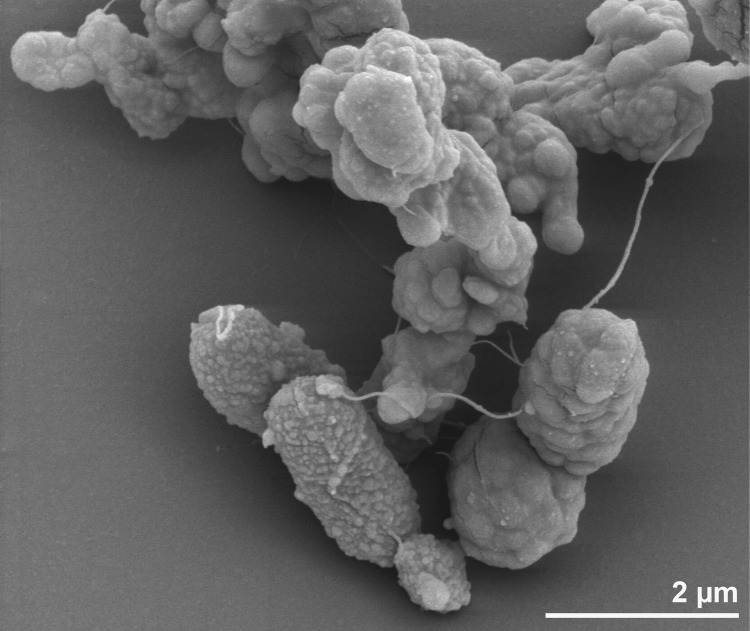
Scanning electron micrograph of *D. thermolithotrophum* BSA^T^

The cells of strain BSA^T^ are small rods, about 1-2 µm long and 0.4-0-5 µm wide and occur singly or in pairs (Figure 2) [1]. Cells stain Gram-negative and are motile *via* three polar flagella; spores are not produced [1]. Strain BSA^T^ grows between 40 and 75°C with an optimum around 70°C, while no growth is detected at 37 or 80°C after 48 h incubation [1]. Growth occurs between pH 4.4 and 8, with an optimum around pH 6.25. No growth is detected at pH 3.7 or 8.5 after 48h incubation at 70°C [1]. Growth is observed in sea salts at concentrations ranging from 15 to 70g/l, with an optimum of approximately 35g/l (corresponding to 23 g NaCl/l [1]). No growth was observed in sea salts at concentrations of 10 and 80 g/l after 48 h incubation at 70°C [1]. Under optimal growth conditions (temperature, pH and NaCl), the doubling time of strain BSA^T^ is around 135 min [1]. Strain BSA^T^ is a strictly anaerobic chemolithotrophic organism that uses sulfur as an electron acceptor in the presence of H^+^ for growth [1]. It utilizes thiosulfate, sulfite and polysulfides as alternative electron acceptors with H_2_ as an electron donor. Cysteine, nitrate or nitrite are not utilized and growth on sulfur, thiosulfate, polysulfides or sulfite was accompanied by exponential H_2_S production [1]. No growth was observed on acetate, formate, methanol, monomethylamine and yeast extract with N_2_-CO_2_ or H_2_ atmosphere in the presence or absence of sulfur [1]. Nitrate, tryptone and yeast extract were used as nitrogen sources [1]. Growth of strain BSA^T^ was inhibited by chloramphenicol, penicillin G and rifampicin at 100 µg/ml but not by streptomycin when added before incubation at the optimum temperature [1].

### Chemotaxonomy

The total lipid content of strain BSA^T^ is about 6% of the total dry weight and is characterized by the presence of aminophospholipids and a phospholipid at about 66%, R*_f_* 0.7 and 30%, R *_f_* 0.5, respectively, as well as minor compounds [1]. Gas chromatographic analysis of fatty acid components of both compounds revealed the presence of saturated and monounsaturated acyl chains [1]. The phosphoinositol contains C_16:0_ (15%), C_18:1_ (41%) identified as methyl-oleate, and C_18:0_ (44%) identified as stearate. The phosphoamino-positive compounds contained C_16:0_ (14%), C_18:1_ (43%), C_18:0_ (31%) and C_20:0_ (12%), as well as minor compounds [1].

## Genome sequencing and annotation

### Genome project history

This organism was selected for sequencing on the basis of its phylogenetic position [31], and is part of the *** G****enomic* ***E****ncyclopedia of* ***B****acteria and* ***A****rchaea * project [32]. The genome project is deposited in the Genomes On Line Database [13] and the complete genome sequence is deposited in GenBank. Sequencing, finishing and annotation were performed by the DOE Joint Genome Institute (JGI). A summary of the project information is shown in Table 2.

**Table 2:** Genome sequencing project information

**MIGS ID**	**Property**	**Term**
MIGS-31	Finishing quality	Finished
MIGS-28	Libraries used	Four genomic libraries: one 454 pyrosequence standard library, two 454 PE library (10.5 kb insert size), one Illumina library
MIGS-29	Sequencing platforms	Illumina GAii, 454 GS FLX Titanium
MIGS-31.2	Sequencing coverage	282.0 × Illumina; 40.0 x pyrosequence
MIGS-30	Assemblers	Newbler version 2.3, p Velvet version 0.7.63, phrap version SPS - 4.24
MIGS-32	Gene calling method	Prodigal 1.4, GenePRIMP
	INSDC ID	CP002543
	Genbank Date of Release	March 2, 2011
	GOLD ID	Gc01671
	NCBI project ID	51497
	Database: IMG-GEBA	2503754020
MIGS-13	Source material identifier	DSM 11699
	Project relevance	Tree of Life, GEBA

### Growth conditions and DNA isolation

*D. thermolithotrophum* strain BSA^T^, DSM 11699, was grown anaerobically in DSMZ medium 829 (*Desulfurobacterium* medium) [34] at 70°C. DNA was isolated from 0.5-1 g of cell paste using Qiagen Genomic 500 DNA Kit (Qiagen 10262) following the standard protocol as recommended by the manufacturer without modifications. DNA is available through the DNA Bank Network [35].

### Genome sequencing and assembly

The genome was sequenced using a combination of Illumina and 454 sequencing platforms. All general aspects of library construction and sequencing can be found at the JGI website [36]. Pyrosequencing reads were assembled using the Newbler assembler (Roche). The initial Newbler assembly consisting of 96 contigs in one scaffold was converted into a phrap [37] assembly by making fake reads from the consensus, to collect the read pairs in the 454 paired end library. Illumina GAii sequencing data (45.0 Mb) was assembled with Velvet [38] and the consensus sequences were shredded into 1.5 kb overlapped fake reads and assembled together with the 454 data. The 454 draft assembly was based on 192.1 Mb 454 draft data and all of the 454 paired end data. Newbler parameters are -consed -a 50 -l 350 -g -m -ml 20. The Phred/Phrap/Consed software package [37] was used for sequence assembly and quality assessment in the subsequent finishing process. After the shotgun stage, reads were assembled with parallel phrap (High Performance Software, LLC). Possible mis-assemblies were corrected with gapResolution [36], Dupfinisher [39], or sequencing cloned bridging PCR fragments with subcloning. Gaps between contigs were closed by editing in Consed, by PCR and by Bubble PCR primer walks (J.-F. Chang, unpublished). A total of 101 additional reactions were necessary to close gaps and to raise the quality of the finished sequence. Illumina reads were also used to correct potential base errors and increase consensus quality using a software Polisher developed at JGI [40]. The error rate of the completed genome sequence is less than 1 in 100,000. Together, the combination of the Illumina and 454 sequencing platforms provided 322.0 × coverage of the genome. The final assembly contained 126,482 pyrosequence and 12,545,740 Illumina reads.

### Genome annotation

Genes were identified using Prodigal [41] as part of the Oak Ridge National Laboratory genome annotation pipeline, followed by a round of manual curation using the JGI GenePRIMP pipeline [42]. The predicted CDSs were translated and used to search the National Center for Biotechnology Information (NCBI) nonredundant database, UniProt, TIGR-Fam, Pfam, PRIAM, KEGG, COG, and InterPro databases. Additional gene prediction analysis and functional annotation was performed within the Integrated Microbial Genomes - Expert Review (IMG-ER) platform [33].

## Genome properties

**Figure 3:**
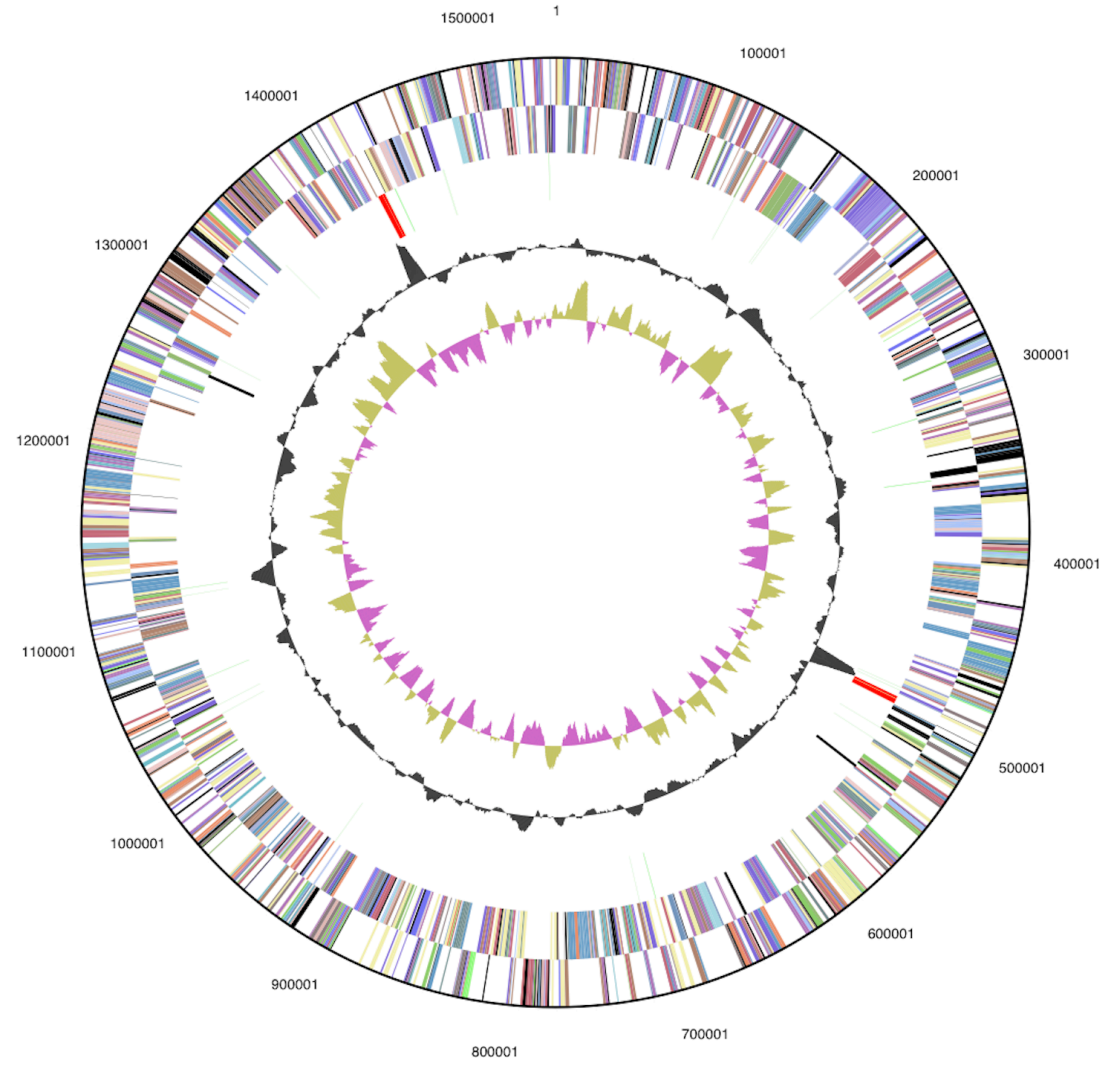
Graphical circular map of the genome. From bottom to top: Genes on forward strand (color by COG categories), Genes on reverse strand (color by COG categories), RNA genes (tRNAs green, rRNAs red, other RNAs black), GC content, GC skew.

The genome consists of one circular chromosome with a total length of 1,541,968 bp and a G+C content of 35.0% (Table 3 and Figure 3). Of the 1,594 genes predicted, 1,543 were protein-coding genes, and 51 RNAs; 34 pseudogenes were also identified. The majority of the protein-coding genes (75.5%) were assigned a putative function while the remaining ones were annotated as hypothetical proteins. The distribution of genes into COGs functional categories is presented in Table 4.

**Table 3:** Genome Statistics

**Attribute**	**Value**	**% of Total**
Genome size (bp)	1,541,968	100.00%
DNA coding region (bp)	1,448,295	93.93%
DNA G+C content (bp)	538,896	34.95%
Number of replicons	1	
Extrachromosomal elements	0	
Total genes	1,594	100.00%
RNA genes	51	3.20%
rRNA operons	2	
tRNA genes	43	2.70%
Protein-coding genes	1,543	96.80%
Pseudo genes	34	2.13%
Genes with function prediction	1,204	75.53%
Genes in paralog clusters	600	37.64%
Genes assigned to COGs	1,330	83.44%
Genes assigned Pfam domains	1,327	83.25%
Genes with signal peptides	394	24.72%
Genes with transmembrane helices	322	20.20%
CRISPR repeats	1	

**Table 4:** Number of genes associated with the general COG functional categories

**Code**	**value**	**%age**	**Description**
J	142	9.7	Translation, ribosomal structure and biogenesis
A	0	0.0	RNA processing and modification
K	47	3.2	Transcription
L	126	8.6	Replication, recombination and repair
B	1	0.1	Chromatin structure and dynamics
D	20	1.4	Cell cycle control, cell division, chromosome partitioning
Y	0	0.0	Nuclear structure
V	13	0.9	Defense mechanisms
T	52	3.6	Signal transduction mechanisms
M	110	7.5	Cell wall/membrane/envelope biogenesis
N	67	4.6	Cell motility
Z	0	0.0	Cytoskeleton
W	0	0.0	Extracellular structures
U	74	5.1	Intracellular trafficking, secretion, and vesicular transport
O	55	3.8	Posttranslational modification, protein turnover, chaperones
C	113	7.7	Energy production and conversion
G	42	2.9	Carbohydrate transport and metabolism
E	108	7.4	Amino acid transport and metabolism
F	57	3.9	Nucleotide transport and metabolism
H	88	6.0	Coenzyme transport and metabolism
I	38	2.6	Lipid transport and metabolism
P	57	3.9	Inorganic ion transport and metabolism
Q	13	0.9	Secondary metabolites biosynthesis, transport and catabolism
R	140	9.6	General function prediction only
S	97	6.6	Function unknown
-	264	16.6	Not in COGs
